# Gigantol Preserves Lens Biophysical Homeostasis by Restoring Cytoskeletal Integrity and Membrane Fluidity in a Diabetic Cataract Model

**DOI:** 10.3390/ijms27020569

**Published:** 2026-01-06

**Authors:** Xue Li, Xinduo Huang, Xiaoyong Wei

**Affiliations:** School of Basic Medical Sciences, Guangzhou University of Chinese Medicine, Guangzhou 510006, China; jidewowbf@163.com (X.L.); jidewowml@163.com (X.H.)

**Keywords:** gigantol, biomechanics, surface morphology, ultrastructure, HLECs, DC

## Abstract

Diabetic cataract (DC) is a major complication of diabetes, with human lens epithelial cells (HLECs) playing a central role in its pathogenesis. Gigantol, a natural compound, has demonstrated protective effects against HLEC damage, yet its underlying mechanisms, particularly concerning cellular biophysical properties, remain poorly understood. This study investigated the protective role of gigantol against high-glucose-induced damage in HLECs, with a specific focus on alterations in cellular biophysical properties. Using a multi-technique approach including transmission electron microscopy (TEM), atomic force microscopy, laser scanning confocal microscopy, and Raman spectroscopy, we analyzed changes in ultrastructure, morphology, stiffness, roughness, membrane fluidity, and cytoskeletal organization. Treatment with gigantol effectively restored cellular ultrastructure, mitigated cytoskeletal disruption, and normalized key biomechanical properties: it reduced cell stiffness and roughness by approximately one-fourth, increased cell height by nearly onefold, and enhanced membrane fluidity by one-fifth. Raman spectroscopy indicated that gigantol improved membrane fluidity by modulating lipid bilayer structure, specifically through alterations in –CH_2_– bending and –C=C– stretching modes. These findings demonstrate that gigantol protects HLECs from high-glucose-induced damage not only by biochemical means but also by restoring cellular biophysical homeostasis. This study provides novel biophysical–pathological insights into the anti-cataract mechanism of gigantol, highlighting its potential as a therapeutic agent that targets both biochemical and biophysical aspects of DC.

## 1. Introduction

As a leading cause of vision impairment among diabetic patients, DC poses a substantial and growing global health challenge, driven by the escalating prevalence of diabetes mellitus [1]. The pathogenesis of DC is multifactorial, involving chronic hyperglycemia-induced oxidative stress, osmotic imbalance, and advanced glycation end-product (AGE) accumulation [2,3,4]. Among the various affected ocular tissues, lens epithelial cells (LECs) play a pivotal role in maintaining lens transparency and homeostasis, including ion balance, oxidative damage repair, and the precise differentiation of lens fiber cells [5]. Any structural or functional impairment in HLECs can lead to loss of transparency and ultimately cataract formation [6]. Consequently, HLECs are a primary target of hyperglycemic injury, and their dysfunction is a seminal event in cataractogenesis [5,6].

Beyond well-characterized biochemical pathways, the field of cellular biophysical properties has emerged as a vital framework for re-examining DC pathology [6,7]. Physical properties including cell stiffness, membrane topography, and lipid bilayer fluidity are now understood to be dynamic determinants of cell health, influencing everything from mechanotransduction signaling to resilience against stress [8]. In DC, hyperglycemia is suspected to disrupt mechanical homeostasis; however, the precise mechanisms and potential for therapeutic intervention remain largely unexplored [9]. For instance, increased cell stiffness has been linked to cytoskeletal disorganization and impaired cellular volume, while reduced membrane fluidity can hinder nutrient uptake and waste expulsion, exacerbating cellular stress [8,10]. In the context of DC, high glucose levels have been shown to alter the biomechanical integrity of LECs, leading to cellular dysfunction and apoptosis [6,9,10,11].

The cytoskeleton, composed primarily of actin filaments (F-actin), microtubules, and intermediate filaments, is a key determinant of cellular biophysics [12,13]. It provides structural support, facilitates cell adhesion and migration, and regulates mechanotransduction pathways [14]. Under diabetic conditions, hyperglycemia induces oxidative stress and activates the polyol pathway, leading to cytoskeletal remodeling and loss of F-actin organization [15]. This disruption not only compromises the mechanical stability of HLECs but also affects their ability to maintain lens transparency [16,17] and fluidity [18]. Several studies have demonstrated that alterations in F-actin architecture are closely associated with posterior capsule opacification and cataractogenesis [19].

Atomic force microscopy (AFM) has emerged as a powerful tool for quantifying the biomechanical properties of living cells at the nanoscale [18,20]. By measuring parameters such as Young’s modulus (stiffness), surface roughness, and cell height, AFM provides insights into the mechanical health of cells under physiological and pathological conditions [21]. In diabetic models, HLECs exhibit increased stiffness and surface roughness, which correlate with elevated oxidative stress and inflammatory markers [22,23]. These changes are indicative of a loss of viscoelasticity and the increased brittleness of the cells, making them more susceptible to damage [24].

Another critical aspect of cellular biophysics is membrane fluidity, which reflects the dynamic state of the lipid bilayer and its embedded proteins [18]. Reduced membrane fluidity, often resulting from lipid peroxidation or changes in phospholipid composition, impairs ion channel function, receptor signaling, and overall cellular resilience [20]. Raman spectroscopy has been widely used to assess membrane fluidity by analyzing the vibrational modes of –CH_2_– and –C–C– bonds in lipid chains. In DC, a decrease in membrane fluidity has been observed [25], which may contribute to the failure of HLECs to cope with metabolic and oxidative insults [26].

Despite advances in understanding the biophysical pathways involved in DC, the biophysical aspects of LEC injury remain underexplored. Most existing therapies focus on antioxidant or osmotic regulation, with limited attention to restoring cellular mechanical properties [27]. This gap highlights the need for novel therapeutic agents that can protect or reverse the biomechanical damage induced by hyperglycemia [28]. Previous studies from our group have utilized techniques such as AFM, laser confocal scanning microscopy (LCSM), and Raman spectroscopy to demonstrate the potential of syringic acid—extracted from Dendrobium—in restoring high-glucose-damaged HLECs and mitigating DC [29].

Gigantol, a bibenzyl compound derived from Dendrobium species, which exhibits a wide range of pharmacological activities including anti-tumor, anti-diabetic nephropathy, anti-hyperuricemia, and anti-inflammatory effects [30,31,32,33], has attracted attention for its anti-cataract properties. Our previous studies have shown that gigantol can inhibit aldose reductase, attenuate oxidative stress, and reduce sorbitol accumulation in the lens [29,34,35,36,37]. However, its effects on the biophysical properties of LECs have not been systematically investigated. Given the central role of cellular mechanics in DC progression, we hypothesize that gigantol protects HLECs from high-glucose-induced damage by restoring cellular biophysical homeostasis, including cytoskeletal integrity and membrane fluidity.

This study aims to bridge this knowledge gap by examining the influence of gigantol on the biophysical properties of HLECs under high-glucose conditions. Using a combination of AFM, TEM, confocal microscopy, and Raman spectroscopy [38,39,40,41,42,43,44,45,46,47,48,49], we sought to characterize changes in HLECs’ stiffness, roughness, thickness, membrane fluidity, and cytoskeletal organization, thereby restoring their structural and functional integrity.

The findings of this study are expected to provide a biophysics perspective on the anti-cataract mechanism of gigantol, highlighting its potential as a multi-target agent that not only addresses biochemical imbalances but also restores cellular mechanical health. Such insights could pave the way for the development of novel biophysically informed therapies for diabetic cataract, offering a complementary approach to existing pharmacological strategies.

## 2. Results

### 2.1. Damaged Ultrastructure Following High-Glucose Stimulation Is Repaired by Gigantol

**Figure 1:**
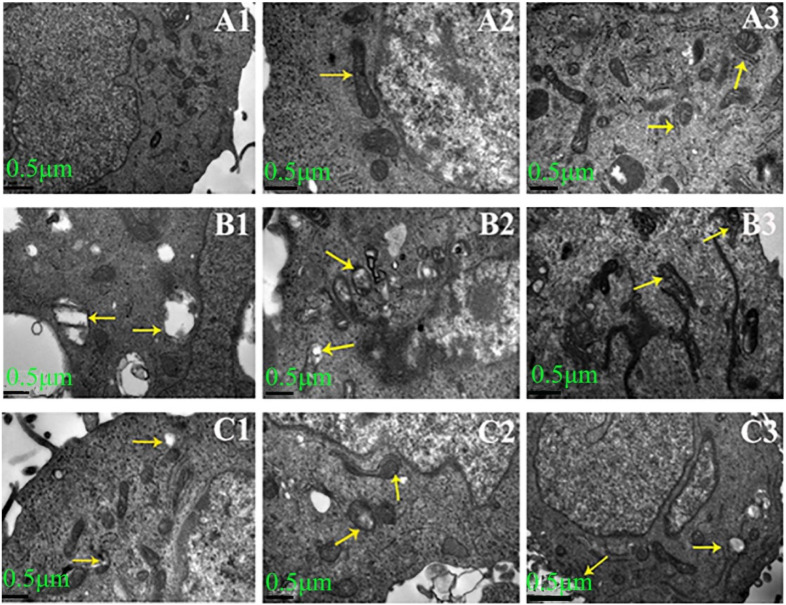
The morphological features of glucose-induced HLECs treated with gigantol under TEM (×12,000). (**A1**–**A3**) in the control group: showing a typical ultrastructure characterized by mitochondria and endoplasmic reticulum in HLECs; (**B1**–**B3**) in the model group: showing the obvious vacuoles (**B1**) and abnormal cristae in mitochondria (**B2**) and endoplasmic reticulum (**B3**) in the cytoplasm; (**C1**–**C3**) in the gigantol group: showing the decreased vacuoles (**C1**) and increased normal cristae in mitochondria (**C2**) and endoplasmic reticulum (**C3**). The significant changes in organelles are pointed out using arrows.

The ultrastructure of HLECs is observed using TEM. In the control group, the cytoplasmic organelles exhibited a typical ultrastructure in mitochondria and endoplasmic reticulum (Figure 1(A1–A3)). In the model group, large vacuoles were present in the cytoplasm and mitochondria, and ribosomes were detached from the endoplasmic reticulum (Figure 1(B2,B3)). In the gigantol group, the damaged ultrastructure was restored, vacuoles in the cytoplasm were reduced, and the endoplasmic reticulum and mitochondria exhibited a relatively complete structure (Figure 1(C1–C3)).

### 2.2. Gigantol Restores Surface Morphology of HLECs

AFM was performed to investigate the surface morphology of HLECs at a high resolution. AFM enables the quantitative assessment of cellular morphology at the single-cell level. The cantilever, by applying a controlled force, can precisely map the surface topography and measure the height of cellular structures. To ascertain the statistical significance, the means of 20 cells were calculated for each dataset based on the recorded AFM images.

**Figure 2:**
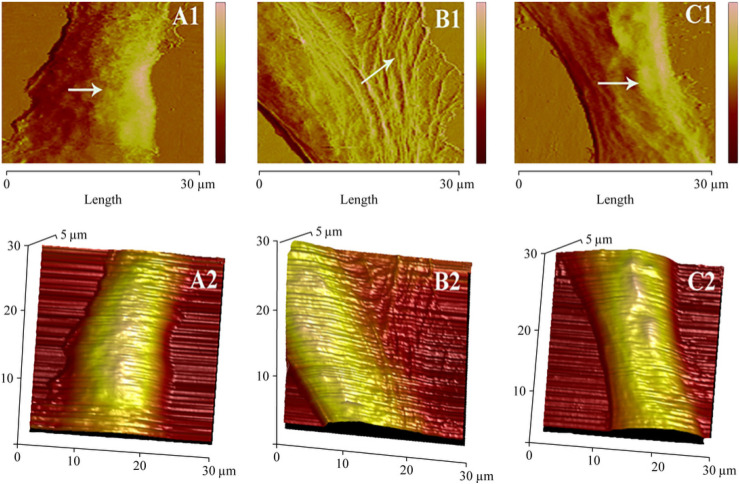
The AFM images of HLECs. Row 1 (**A1**,**B1**,**C1**) shows the surface morphology images of HLECs, Row 2 (**A2**,**B2**,**C2**) displays the 3-D height images. The normal group, model group, and gigantol group are presented in columns 1–3 for each HLEC. White arrows in (**A1**,**B1**,**C1**) indicate the difference in cell membranes between the three groups.

Untreated HLECs in the control group exhibited smooth and plump cell shapes with clear edges (Figure 2(A1)). In the model group, treated with 50 mM glucose, the surface morphology of HLECs was highly folded with edges spreading outward compared to the control group (Figure 2(B1)), suggesting the existence of a stress response, which was decreased by gigantol treatment. HLEC membrane appeared to be relatively unperturbed, exhibiting only slight deformations (Figure 2(C1)). The subsequent 3-D magnification (Figure 2(A2,B2,C2)) further emphasizes this observation.

**Figure 3:**
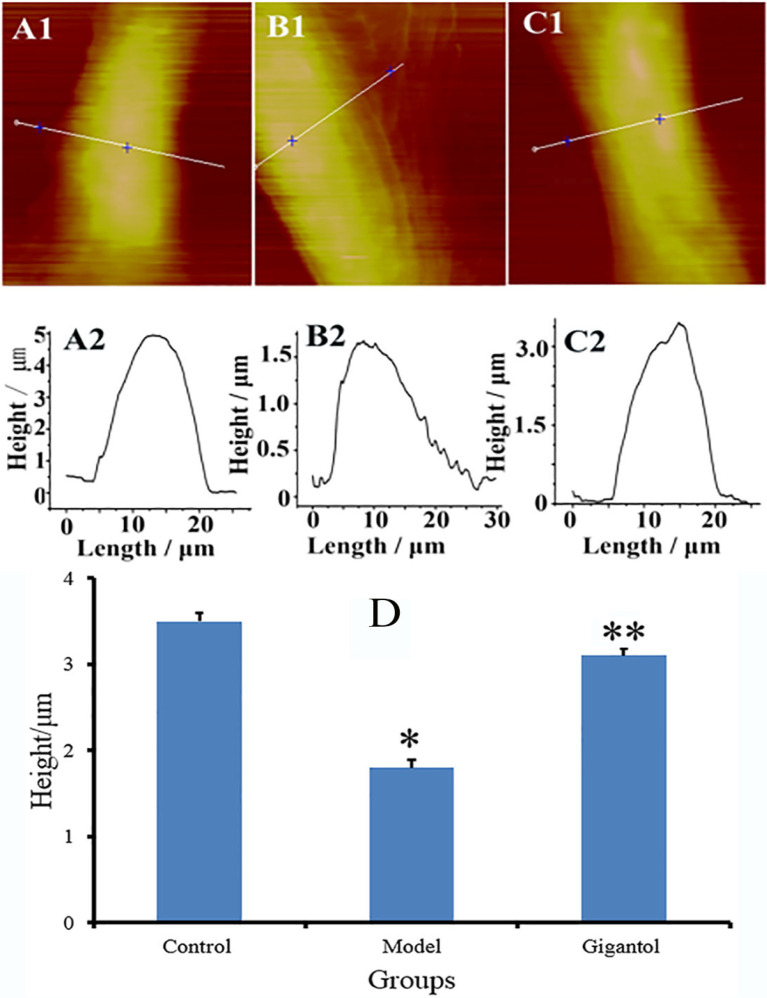
The AFM height images of HLECs. The images of the control, model, and gigantol groups are presented in columns 1–3 for each HLEC. Row 1 (**A1**,**B1**,**C1**) depicts the height images of the HLEC from the deflection error image, and row 2 (**A2**,**B2**,**C2**) depicts the height profiles obtained from the section line as marked in row 1. Row 3 the histogram (**D**) displays the average height of cells for each group. * *p*  <  0.01, the model group compared to the control group; ** *p*  <  0.01, the gigantol group compared to the model group.

The cell height (Figure 3) further corroborates the restoration of cellular morphology. A cross-sectional deformation profile revealed the height of HLECs to be 3.5 ± 0.35 μm, 1.8 ± 0.25 μm, and 3.1 ± 0.15 μm in the control, model, and gigantol groups, respectively (Figure 3(A2,B2,C2)). Gigantol could partially restore the height of HLECs in the presence of high glucose (*p* < 0.01, Figure 3D).

### 2.3. Gigantol Decreases Surface Roughness of HLECs in High-Glucose Medium

**Figure 4:**
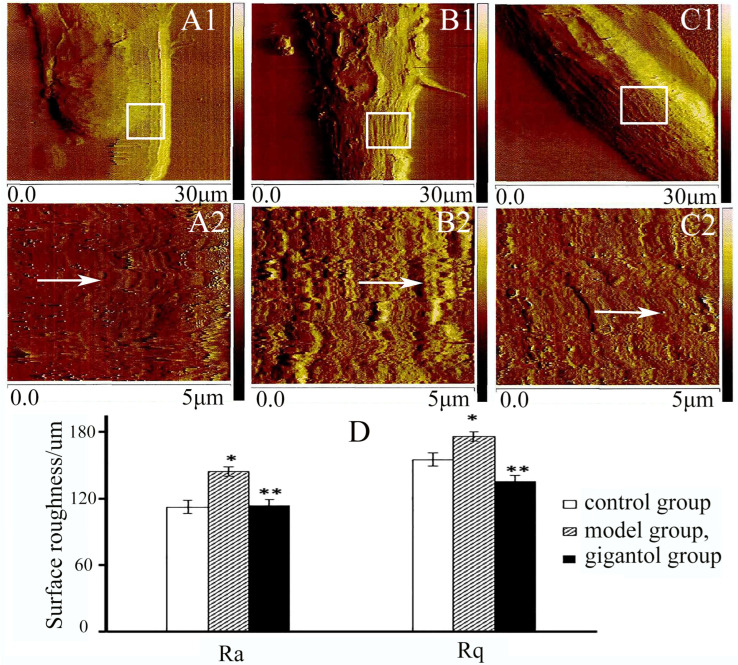
HLEC roughness. The images of the control, model, and gigantol groups are presented in columns 1–3 for each HLEC. Row 1 (**A1**,**B1**,**C1**) depicts the AFM images of the HLEC, with a scanned area of 30 μm  × 30 μm, and row 2 (**A2**,**B2**,**C2**) depicts the enlarged images, with a scanned area of 5 μm  ×  5 μm, as white box areas marked in row 1 (**A1**,**B1**,**C1**). Moreover, the average Ra and Rq of HLEC for each group to illustrate the roughness parameters are shown in the histogram (**D**). White arrows in (**A2**,**B2**,**C2**) indicate the difference in the fold and particles of the cell membrane among the three groups. * *p*  <  0.01, the model group compared to the control group; ** *p*  <  0.05 the gigantol group compared to the model group.

The fine details of the HLEC surface structure were visualized using AFM peak force error images (Figure 4). The apparent changes were noted on the surfaces of the HLECs. In the control group, a smooth and well-defined surface was exhibited by HLECs (Figure 4(A1,A2)); in the model group, the clear and distinct fold and convex particles of the cell membrane were observed (Figure 4(B1,B2)); in the gigantol group, little streaks existed on the cell surface, and the cell was well defined (Figure 4(C1,C2)).

The Ra and Rq were measured within a 5 μm × 5 μm local region selected from the AFM peak force error images (Figure 4(A2,B2,C2)). The quantitative analysis of AFM topographical data revealed that high-glucose stimulation significantly altered the surface architecture of HLECs. The Ra increased from 112.25 ± 2.01 nm in the control group to 144.2 ± 4.13 nm in the model group (*p* < 0.01). Notably, gigantol markedly attenuated this effect, bringing the Ra value down to 114 ± 3.45 nm (*p* < 0.01) (Figure 4D). A similar trend was observed for the Rq, which was normalized by gigantol from 155 ± 3.07 nm (control group) and 176.6 ± 3.54 nm (model group) to 135 ± 5.12 nm (*p* < 0.05) (Figure 4D).

### 2.4. Gigantol Reduces Stiffness of HLECs in High-Glucose Conditions

**Figure 5:**
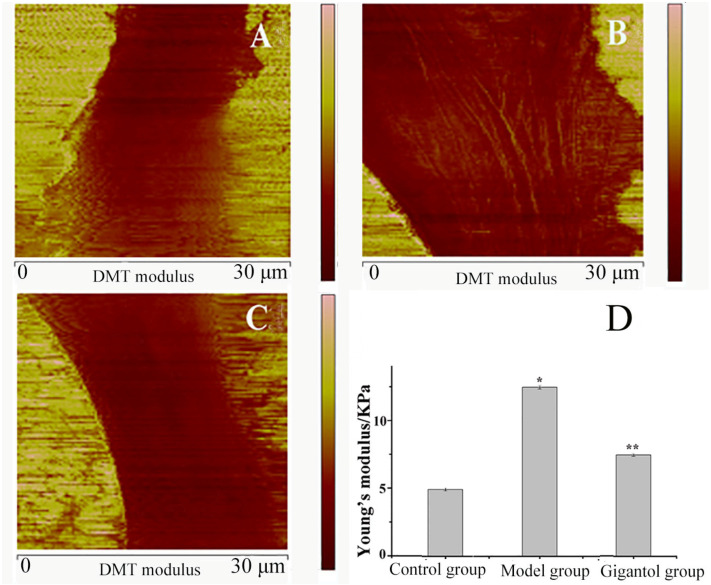
The AFM topographic images of HLEC stiffness. (**A**), (**B**), and (**C**) are the DMT modulus images of the control, model, and gigantol groups, respectively. The average Young’s modulus of cells for each group is shown in (**D**). * *p*  <  0.01, the model group compared to the control group; ** *p*  <  0.01, the gigantol group compared to the model group.

Cell stiffness was investigated by monitoring changes in Young’s modulus. The Young’s modulus was estimated by selecting local regions of 5 μm × 5 μm from the AFM of DMT (Derjaguin–Muller–Toporov) images (Figure 5A–C). An AFM-based stiffness assessment showed a Young’s modulus of 5.7 ± 0.56 KPa under control conditions, which increased to 11.6 ± 0.91 KPa in high-glucose conditions, and was partially restored to 7.5 ± 0.45 KPa with gigantol treatment (Figure 5D). The model group exhibited a significant increase in stiffness when compared with the control group (*p* < 0.01). However, after gigantol treatment, the stiffness of all tested HLECs demonstrated a decrease of approximately one-third when compared with the model group (*p* < 0.01).

### 2.5. Cytoskeletal Disruptions Are Alleviated Following Treatment with Gigantol

**Figure 6:**
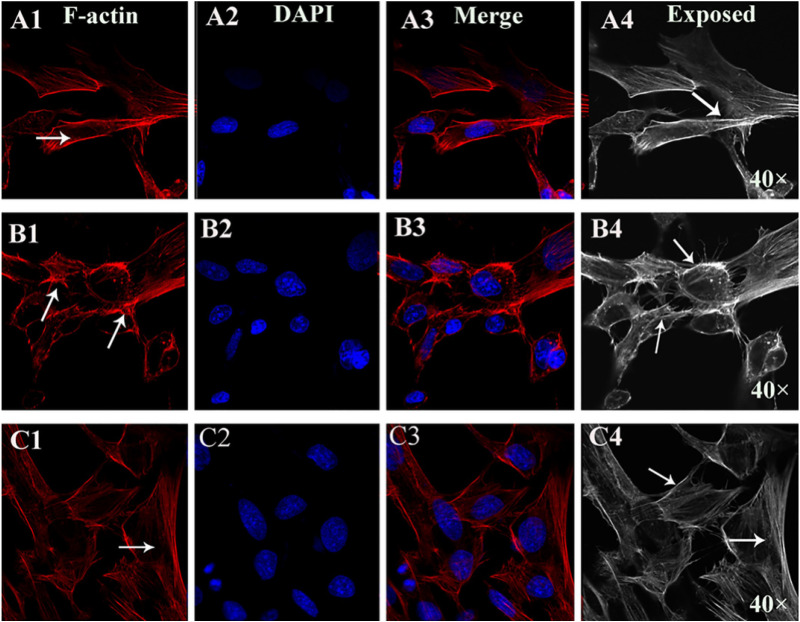
The effects of gigantol on F-actin expression in HLECs. The LSCM images of F-actin, DAPI, merged F-actin and DAPI, and exposed F-actin are presented in columns 1–4 for each group. Rows 1 (**A1**–**A4**), 2 (**B1**–**B4**), and 3 (**C1**–**C4**) depict the control, model, and gigantol groups, respectively (40×  objective, 1.25 NA). White arrows in (**A1**,**B1**,**C1**,**A4**,**B4**,**C4**) indicate the difference the exposed F-actin.

The confocal imaging of F-actin revealed a stark contrast in cytoskeletal architecture across groups following 24 h treatment with gigantol (Figure 6). The control HLECs displayed a characteristic, radially organized network of continuous F-actin (Figure 6(A1–A4)). In stark contrast, a high glucose exposure triggered severe cytoskeletal disarray, manifesting as fragmented, disorganized, and clumped F-actin aggregates (Figure 6(B1–B4)). Gigantol intervention effectively preserved the cytoskeletal integrity, with a notable reappearance of the continuous, filamentous F-actin structure, indicating a restoration of normal cytoarchitecture (Figure 6(C1–C4)).

### 2.6. Evaluation of Membrane Fluidity in Viable HLECs Through S_trans_ and S_lat_ Following Gigantol Treatment

**Figure 7:**
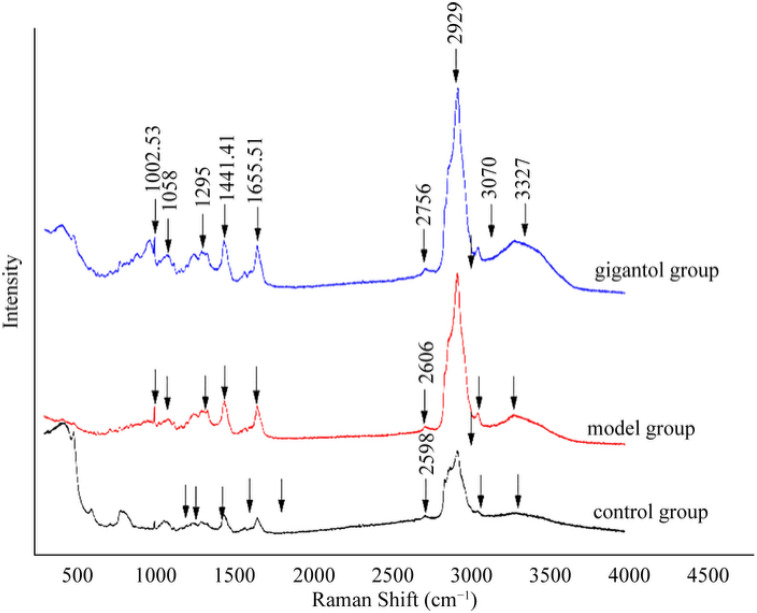
Changes in Raman spectra before and after gigantol treatment for 48 h. The three groups of this experiment: the control group, model group, and gigantol group.

Raman spectroscopy provides strong evidence of the structure of the carbon chains of phospholipids of the cell membrane, which include the order parameter changes in the lateral interaction between chains and the longitudinal interactions within the chains, as well as the changes in the trans and gauche conformations of the hydrocarbon chains. Raman spectroscopy has already been used to detect cell membrane fluidity [49]. The structural changes in the carbon chains of phospholipids of the cell membrane modified with gigantol were observed using Raman spectra in this study. The characteristic peaks of the phospholipids in the Raman spectra mainly included the –CH– stretch vibrations (2900–3000 cm^−1^) and the –C–C– stretch vibrations (1000–1200 cm^−1^) (Figure 7).

In the Raman lines, the major Raman peaks were present at 1006 cm^−1^, 1012 cm^−1^, 1055 cm^−1^, 1089 cm^−1^, 1130 cm^−1^, 1205 cm^−1^, 1448 cm^−1^, 1308 cm^−1^, 1660 cm^−1^, 2778 cm^−1^, and 2937 cm^−1^ (Table 1). Furthermore, the absolute intensities of Raman spectra at 1441.41 cm^−1^ and 1655.51 cm^−1^ were increased in the gigantol group when compared with the control and model groups (Figure 7). Raman lines exhibiting a value of 1448 cm^−1^ (Figure 7) were attributed to the –CH_2_– bending mode, and those with a value of 1660 cm^−1^ (Figure 7) were attributed to the –C=C– stretching mode. From the results, the rates of the S_t_ and S_l_ parameters in the model group were increased by 25.10% and 24.60% (Table 2) when compared with the control group, respectively, whereas the alteration rates of the S_t_ and S_l_ parameters in the gigantol group were all decreased by 19.56% (Table 2) when compared with the model group, respectively. The values of parameters S_t_ and S_l_ in the model group indicated the increase in trans conformation and the decrease in gauche conformation in the lipid bilayer of HLECs, which resulted in the increase in the longitudinal order of the carbon chain. This increase revealed that the liquidity of the cytolemma was decreased. In contrast to the model group, the change in parameters S_t_ and S_l_ in the gigantol group indicated the decrease in trans conformation and the increase in gauche conformation in the lipid bilayer of viable HLECs, which resulted in the longitudinal decrease in the order of the carbon chain and suggested that the liquidity of the cytolemma was increased. Taken collectively, the data indicate that gigantol could increase the liquidity of the cytolemma in the model of glucose-induced HLECs via the induction of alterations in the structure (–CH_2_– bending mode and –C=C– stretching mode) of the lipid bilayer in HLECs.

## 3. Discussion

The pathogenesis of DC has been predominantly investigated through the lens of biochemistry, focusing on pathways such as the polyol cascade, oxidative stress, and the advanced glycation end-product formation. While these mechanisms are indisputably important, they represent only one dimension of a multifaceted disease process. Our study introduces a paradigm shift by demonstrating that profound biophysical dysfunction is a central, hitherto underappreciated, component of high-glucose-induced injury in HLECs. We provide compelling evidence that gigantol exerts its potent cytoprotective effects primarily by acting as a multi-target biomechanical modulator, effectively rescuing HLECs from a state of mechanical failure. This reconceptualization of gigantol’s action—from a conventional antioxidant to a “mechano-therapeutic” agent—represents the cornerstone of our work’s novelty.

A healthy HLEC maintains lens transparency and homeostasis not only through biochemical equilibrium but also through a specific set of biophysical properties—including appropriate cell shape, membrane fluidity, and cytoskeletal compliance [50]. Our data reveal that a high glucose exposure catastrophically disrupts this biophysical homeostasis, driving the cell into a state of aberrant stiffness and structural disorder, which we term the “diabetic mechanical phenotype”.

The most direct evidence is provided by AFM, which quantified a near doubling of the Young’s modulus in HLECs under high-glucose conditions, indicating a significant increase in cell stiffness. Cell stiffness is predominantly governed by the actin cortex—a dense, cross-linked network of F-actin and associated proteins beneath the plasma membrane [51]. The significant reduction in cell stiffness following gigantol treatment, bringing it close to control levels, is a pivotal finding. This “softening” effect is directly linked to the restoration of a more physiological, ordered F-actin network observed by LSCM [52,53,54]. Our confocal microscopy results provide the structural correlate for this mechanical measurement: the disintegration of the native, radially organized F-actin network into disorganized, aggregated clusters. This pathological remodeling is characteristic of sustained actomyosin contractility, frequently driven by the hyperactivation of the RhoA/ROCK signaling pathway, a well-documented phenomenon in diabetic complications [55]. Many naturally derived polyphenols have been shown to inhibit the RhoA/ROCK pathway [56]. By inhibiting ROCK, gigantol could decrease the phosphorylation of the myosin light chain, thereby reducing actomyosin contractility and leading to the dissolution of stress fibers and a more compliant cellular state [57]. Gigantol appears to disrupt the cycle of pathological actin polymerization and hypercontraction, likely by modulating the key regulators of actin dynamics. The restoration of cell height and the smoothing of the cell surface are natural biophysical consequences of this cytoskeletal relaxation and re-organization, as the cell loses its hypercontracted, stressed morphology. This mechanical hardening is not a passive bystander effect; rather, it actively disrupts essential cellular processes [58,59,60,61,62]. In high-glucose conditions, increased intracellular stiffness can impair the trafficking of vesicles and organelles, alter nuclear mechanotransduction, leading to aberrant gene expression, and promote a pro-fibrotic phenotype, a key step in cataractogenesis [63,64,65].

Concurrently, we observed a significant increase in cell surface roughness under high-glucose conditions. Surface roughness is a sensitive indicator of disruptions at the membrane–cytoskeleton interface. Proteins like ezrin, radixin, and moesin (ERM) tether the plasma membrane to the underlying actin cortex [66,67]. A disorganized cortex leads to the failure of these tethers, causing the membrane to bleb or form irregular protrusions and thereby increasing roughness—a process driven by non-muscle myosin II activity along newly formed, rigid actin stress fibers [68].

Beyond the cytoskeleton, our Raman spectroscopy data uncovered a critical mechanical defect at the molecular level: a severe reduction in membrane fluidity. The elevated S_lat_ and S_trans_ order parameters indicate that the lipid acyl chains in the plasma membrane adopted a more extended, rigid trans conformation and packed more tightly, severely restricting the freedom of motion for individual lipid molecules [69]. This “membrane rigidification” is a known consequence of lipid peroxidation and the formation of advanced glycation end-products on membrane components—both of which are exacerbated in a hyperglycemic environment [70,71]. A rigidified membrane impairs the function of embedded ion channels and transporters, hampers the assembly and dynamics of signaling complexes in lipid rafts, and reduces the cell’s ability to repair its membrane after injury [72]. Thus, high glucose orchestrates a mechanical crisis on dual fronts: it stiffens the cytoskeletal core and rigidifies the lipid envelope, creating a functionally compromised cell primed for apoptosis and cataract formation. This hypothesis is strongly supported by observed shifts in the –CH_2_– bending and –C=C– stretching modes in the Raman spectra, which are highly sensitive indicators of lipid chain conformation, packing density, and order [58,59,60,61,62]. This membrane-fluidizing effect is pharmacologically significant. It can restore the proper function of membrane proteins such as transporters and receptors, facilitate nutrient uptake, and improve the cell’s resilience to osmotic stress [73,74]. Furthermore, a more fluid membrane can better distribute mechanical forces and interact dynamically with the underlying cytoskeleton, promoting overall mechanical coherence and integrity [75,76,77].

Gigantol presents an integrated mechano-pharmacological synergy in cytoprotection. The power and novelty of gigantol’s action lie in the synergistic interplay between its effects on the cytoskeleton and the plasma membrane. These are not two isolated compartments but are intimately coupled through a process of cytoprotection feedback [78,79]. A softer, more dynamic cytoskeleton reduces the inward tensile stress on the membrane, allowing it to adopt a more natural, fluid state. A more fluid membrane improves the mobility and function of adhesion complexes and signaling molecules that regulate cytoskeletal dynamics [80,81,82]. Gigantol, by targeting both processes, initiates a positive feedback loop that breaks the vicious cycle of mechanical deterioration initiated by high glucose. The functional benefits of this biomechanical rescue are profound. The restoration of ultrastructural integrity in the gigantol-treated group is likely a downstream effect of this improved mechanical and metabolic homeostasis. A cell with a compliant cytoskeleton and a fluid membrane is better equipped to manage osmotic gradients, distribute organelle-damaging stresses, and maintain efficient mitochondrial function and energy metabolism [83,84,85,86,87]. The recovered cell morphology and mechanics also have critical implications for preventing posterior capsule opacification (PCO) [88,89], a common post-cataract-surgery complication driven by aberrant LEC migration, proliferation, and fibrosis [90,91,92,93,94,95]. The migratory and fibrotic potential of cells is intimately linked to their stiffness and contractility; a gigantol-softened HLEC would be mechanically less prone to pathological epithelial–mesenchymal transition (EMT) and excessive migration, suggesting a broader therapeutic application [78,96,97,98,99,100,101].

Due to its two active hydroxyl and two methoxy functional groups, gigantol exhibits antioxidant and anti-hyperosmotic bioactivities, which underlie its broad pharmacological effects, including anti-tumor, anti-aging, antioxidant, and anti-inflammatory properties. Studies have shown that gigantol exerts anti-hyperuricemic effects through the inhibition of xanthine oxidase activity [32]. It has also been demonstrated to alleviate oxidative stress and inflammation in hepatocytes, inhibit terminal complement complex (TCC) formation, and thereby preserve hepatocyte morphology, indicating hepatoprotective effects [33]. Furthermore, gigantol has been reported to inhibit lung cancer cell proliferation by inducing ubiquitin–proteasomal degradation [30] and attenuate diabetic nephropathy via the suppression of the ROS/MAPK/NF-κB signaling pathway [31]. These findings support our observation that gigantol helps maintain the biochemical properties of lens epithelial cells.

This study reveals that gigantol significantly restores the biomechanical properties of high-glucose-damaged HLECs, with particularly notable efficacy in recovering membrane fluidity. Compared with syringic acid—another bioactive compound from Dendrobium previously reported by our group—both compounds share the ability to ameliorate cellular biophysical dysfunction under diabetic conditions. However, gigantol exhibits distinct and superior mechanisms of action, underscoring the originality of this study’s findings. Specifically, gigantol demonstrates dual-enzyme inhibitory activity: it effectively suppresses aldose reductase (AR), thereby mitigating hyperosmotic stress induced by the polyol pathway, while also significantly inhibiting inducible nitric oxide synthase (iNOS), reducing oxidative and nitrosative damage. In contrast, syringic acid primarily acts through AR inhibition to counter hyperosmotic stress but shows no substantial effect on iNOS activity [29]. This mechanistic divergence translates into a broader functional profile for gigantol: it possesses both anti-hyperosmotic and antioxidant capacities, addressing not only osmotic imbalance-induced alterations in cell morphology and membrane fluidity but also direct structural damage caused by reactive oxygen and nitrogen species. However, syringic acid is largely limited to the anti-hyperosmotic action. Consequently, in restoring glucose-impaired biophysical properties, gigantol’s dual targeting of AR and iNOS allows for earlier and more comprehensive intervention at the biochemical level, resulting in the more pronounced recovery of membrane fluidity, cellular elasticity, and surface topography. This multi-target, dual-functional characteristic not only expands our understanding of the pharmacological diversity of Dendrobium bioactive compounds but also provides new experimental support for developing multi-mechanistic therapeutic strategies against DC. These findings highlight the innovative value of this study in elucidating the unique bioactivity of gigantol.

In summary, our findings confirm that gigantol protects HLECs from high-glucose-induced damage by restoring cellular biomechanical homeostasis, including cytoskeletal integrity and membrane fluidity. The cytoskeleton acts as a cellular stabilizer. High glucose causes F-actin aggregation, disrupts the microfilament network, increases HLEC permeability, and compromises the cellular barrier. This alters cell morphology, stiffness, roughness, viscoelasticity, membrane fluidity, and Young’s modulus and induces trans–gauche isomerization in membrane lipid chains, culminating in cataract formation. Gigantol inhibits the expression and activity of aldose reductase and inducible nitric oxide synthase in HLECs [29,34,35,36,37], restores intracellular osmotic and redox homeostasis, promotes F-actin depolymerization, and enhances its orderliness. Consequently, it restores the hydrocarbon chain structure of phospholipids in the cell membrane, restores the ultrastructure of HLECs under high-glucose conditions, reduces cell surface roughness and stiffness, restores cell surface morphology, alters cell adhesion and migration capabilities, increases membrane fluidity by restoring CH_2_ and C-C structural integrity, and enhances the orderliness of cytoskeletal protein fibers. These actions collectively maintain normal HLEC morphology under high-glucose conditions and exert anti-DC effects.

Based on the potent anti-DC activity of gigantol demonstrated in this study, future work will focus on developing a gigantol-based eye drop formulation for clinical application, aiming to provide a novel therapeutic option for patients with DC.

## 4. Materials and Methods

### 4.1. Cell Culture and Treatment Groups

The HLEC line SRA01/04 was a kind gift from the Ophthalmology Center of the Sun Yat-Sen University (China). SRA01/04 culture procedures followed our previously published protocols [29]. After cells reached 80% confluence, cells were digested with trypsin and used for experiments. The HLECs were randomly allocated into three experimental cohorts (*n* = 5): (1) the control group: maintained in standard MEM (Gibco, Grand Island, NY, USA); supplemented with 10% FBS (Biological Industries, Beit HaEmek, Israel); (2) the model group: exposed to MEM containing 50 mM glucose (Aladdin Industrial Corporation, Shanghai, China) and 10% FBS to simulate diabetic conditions; and (3) the gigantol group: co-treated with 50 mM glucose and 1 μg/mL gigantol (gigantol was kindly provided by the Kunming Institute of Botany, Chinese Academy of Sciences, China, purity ≥98%) in complete MEM.

### 4.2. Transmission Electron Microscopy

HLECs were seeded into 60 mm culture plates at a density of 5  ×  10^4^ cells/well. After treatment for 24 h, cells were collected for TEM. Cells were washed twice with PBS (Gibco, Grand Island, NY, USA) and fixed overnight at 4 °C in 2.5% glutaraldehyde (Sigma-Aldrich, St. Louis, MO, USA). After fixation, they were washed three times with PBS and then post-fixed with 1% osmium tetroxide (Sigma-Aldrich, St. Louis, MO, USA) for 2 h at 4 °C. Samples were dehydrated with gradient ethanol and embedded in epon resin and polymerized at 60 °C for 48 h. The blocks were ultrathin-sectioned at 80 to 100 nm with a diamond knife using an ultramicrotome (CM1860 UV; Leica, Wetzlar, Germany). Ultrathin sections were stained with uranyl acetate for 20 min and lead citrate for 12 min. Images were captured using a transmission electron microscopy (Tecnai G2 Spirit Twin; FEI, Brno, Czech Republic) and then analyzed by the software ImageJ 1.52a software (National Institutes of Health; Bethesda, MD, USA) to measure the ratio of cristae number to mitochondrial area (µm^2^) [38,102,103].

### 4.3. Atomic Force Microscopy

AFM studies were performed in three independent experiments, following previously published protocols [39,104] with slight modifications. All AFM measurements were performed on a Bruker AFM (Multimode 8, Bruker, Santa Barbara, CA, USA), including imaging and force distance measurement. AFM imaging was conducted in the tapping mode in air using an MLm LCT silicon nitride probe with a tip radius of 20 nm. Before and after the measurements, the triangular cantilever was cleaned with EtOH and acetone. After system stabilization for 0.5–1 h, the sample coverslip was rinsed with PBS, and the medium was replaced with FBS-free DMEM to minimize interference. The sample was mounted, and the laser alignment and quadrant photodetector were optimized. Sensitivity and the spring constant were calibrated by the thermal tune method using the equipartition theorem, preceded by continuous ramping. After calibration, the spring constant was set to 0.4017 N/m for performing measurements. Imaging parameters were set with an overall scan size of 30 μm × 50 μm, local high-resolution scans of 5 μm × 5 μm, a Z-range of ±1.5 μm, an approach velocity of 0.5 μm/s, and a resolution of 128 × 128 pixels. To ensure cell viability, the observation time for each group was limited to 1–2 h. Following image acquisition, raw topography data were flattened using the Nanoscope Analysis v 3.0 software (Bruker, Santa Barbara, CA, USA). Subsequently, these processed images were analyzed with ImageJ to quantify specific parameters, including cell height, surface roughness, and Young’s modulus.

First, cell morphology was observed and distinguished. HLECs were grown at a cell density of 5  ×  10^5^ on a six-well plate with glass coverslips pretreated with 0.2% gelatin. Next, cells were treated with gigantol or glucose in different groups for 24 h. Afterwards, cells were washed with PBS and fixed with a 2.5% glutaraldehyde solution for 30 min. Subsequently, the cells were washed with PBS, air-dried, and imaged using AFM, with the following parameters used for the measurements: oxidized sharpened silicon tips with a tip radius of 6 nm; resonant frequency = 190 kHz; spring constant = 45 N/m; scanning speed  =  0.2 Hz; cantilever elastic coefficient  =  1–3 N/M; approach velocity  = 1.0 Hz; and scan size  =  30–50 μm^2^ per cell. Cells were observed for 1–2 h in each group, during which cells remained viable as previously described [40]. Surface roughness and stiffness were assessed using cell surface topography images, with 10–15 areas of 5  ×  5 μm. Imaging parameters were adjusted to minimize the force applied during the scanning of the topography of the samples. The images of the cells were obtained and processed through the nanoscope analysis software.

Second, the surface roughness and shapes of the cell were estimated using the nanoscope analysis software after applying a mean filter to the raw images. A total of 20 measurements were taken on cells for each parameter, providing enough values for statistical evaluation. By using the following equations, the surface roughness of a selected area of this flattened area was calculated from the standard deviation in the height image. The degree of roughness was assessed using the arithmetic mean roughness (R_a_) and the root mean square roughness (R_q_). The R_a_ and R_q_ were calculated using Formulas (1) and (2), respectively.

(1) $$R_{a}=\frac{1}{N}\sum_{j=1}^{N}\left|Z_{j}\right|$$

(2) $$R_{q}=\sqrt{\frac{\sum Z_{i}^{2}}{N}}$$

where N denotes the sampling point, and Z denotes the height of the Z axis [41].

After the position of the cell was determined, force spectroscopy was performed. The measurement of the central region of cell was performed with an approach speed of 10 µm/s and a maximum force of 2 nN. A curve length of 1 µm and a sampling rate of 2 kHz were used. Ten to fifteen cells per sample were measured. The stiffness of each cell was determined by the Young’s modulus [42,105]. The Young’s modulus of the cells was calculated using Hertz’s contact model [43,44]. Initially, the relationships between indentation, denoted as δ, and loading force, denoted as F in the spherical probe, were described by Formula (3) [45], where R represents the radius of the tip and E_r_ represents the reduced Young’s modulus. A reduced Young’s modulus E_r_ was correlated with the Young’s modulus of simple E_s_, which is described by Formula (4) [45]. V_T_ and vs. denote the Poisson ratios of the tips and samples. The Poisson ratio of the cell was assumed to be 0.5 [46].

(3) $$F_{\left(\delta \right)}=\frac{4}{3}\sqrt{R}E_{r}\delta^{3/2}$$

(4) $$\frac{1}{E_{r}}=\frac{1-V_{t}^{2}}{E_{t}}=\frac{1-V_{s}^{2}}{E_{s}}$$

### 4.4. Laser Scanning Confocal Microscopy

The morphology of the HLECs seeded in the wells and on the collagen scaffolds was visualized using laser confocal scanning microscopy (LCSM, LSM 710, Carl Zeiss, Oberkochen, Germany) after 24 and 48 h of treatment. Cell nuclei were stained with 4′,6-diamidino-2-phenylindole (DAPI; Invitrogen, Waltham, MA, USA) and the cytoplasm with rhodamine (Enzo Life Sciences, Farmingdale, NY, USA). F-actin was detected with immunofluorescence.

The cells were fixed with 4% *v*/*v* paraformaldehyde at room temperature for 30 min, rinsed three times with PBS for 5 min in each wash, and permeabilized using 0.1% Triton X-100 (Dingguo Changsheng biotech Co., Ltd., Beijing, China) for 5 min. After permeabilization, the samples were washed again with PBS 3 times for 5 min each. F-actin in the cytoskeleton was stained with rhodamine (5 μM, Cytoskeleton Inc., Denver, CO, USA) for 1 h and then treated with 1% bovine serum albumin (BSA) (Sigma-Aldrich, St. Louis, MO, USA) blocking solution in PBS for 30 min, rinsed 3 times with PBS for 5 min in each wash, stained with 1.5 μM DAPI for 10 min, and then rinsed times with PBS for 5 min in each wash. After the staining procedure and washing, excess moisture was removed using filter paper, Finally, the samples were mounted on microscope slides using 95% glycerol. Fluorescence images were acquired using LCSM with excitation at 360 nm and emission at 440 nm.

### 4.5. Surface-Enhanced Raman Spectroscopy

HLECs were collected using centrifugation at 3000× *g* for 5 min after treatment for 48 h, washed three times, re-suspended in 50 μL PBS, and then dropped onto a quartz slide, and the samples were air-dried for 5 min and visualized using Raman scattering spectra. The spectra were collected using a LabRAM Aramis micro Raman spectrometer (Horiba Jobin Yvon S.A.S., Palaiseau, France). The Raman spectra were collected using the 633 nm excitation laser, with a spot diameter of 1.5 μm and a spatial resolution of 500 nm. A Raman spectral range of 0–4000 cm^−1^ and 10–15 spectra per group were used for the Raman spectroscopy as previously described [46]. The testing time was 30 s for each sample (three times per sample). An integration time of 90 s and a low laser power (50%) were used for spectral acquisition to avoid cellular damage [47]. The Raman spectrum was baseline-corrected, and the Raman intensities were measured as the peak height.

S_l_ refers to the relative intensity of the 2880 cm^−1^ Raman band, which is related to the vibrational coupling between the adjacent chains and gives the semiquantitative measurement of the lateral interactions between –CH_2_– chains. S_t_ refers to the relative intensity of the 1130 cm^−1^ Raman band, which is related to the average number of “trans” bonds in the –C=C– chain and gives the measurement of the longitudinal order of the intrachain structure. The S_t_ and S_l_ of the membranes were used to further investigate the fluidity of HLECs following gigantol treatment. The parameters S_l_ and S_t_ were calculated as follows [48,49]:

(5) $$S_{l}=\frac{I_{CH2}-0.7}{1.5}$$

I_CH2_ = I_2890_/I_2850_ (6)

(7) $$S_{t}=IC=C/1.77$$

I_C=C_ = I_1130_/I_1090_ (8)

where I_2890_/I_2850_ denotes the height ratio of the peaks at 2890 cm^−1^ and 2850 cm^−1^, I_2890_ and I_2850_ represent the symmetrical stretching vibrations and anti-symmetric stretching vibrations of CH_2_ in the cell membrane structures, I_1130_/I_1090_ denotes the height ratio of the peaks at 1130 cm^−1^ and 1090 cm^−1^, and I_1130_, and I_1090_ represent the anti-conformation and twisted-conformation of the framework C-C in the membrane phospholipids, respectively.

### 4.6. Statistical Analysis

All data are presented as the mean ± SD for at least three independent experiments. The software SPSS 22.0 was used to analyze variance. Comparisons between groups were carried out using the unpaired two-tailed Student’s *t*-test and one-way analysis of variance (ANOVA). Differences were considered statistically significant when *p*-values were lower than 0.05 (*p* < 0.05).

## 5. Conclusions

This study pioneers a biomechanical framework for understanding both DC pathology and gigantol’s therapeutic action. We have delineated a clear pathway from high-glucose stress to a state of “mechano-pathology” characterized by cytoskeletal rigidity and membrane rigidification. We further demonstrated that gigantol acts as a potent “mechano-therapeutic” that effectively reverses these defects, restoring mechanical homeostasis and preserving cell viability. This new perspective positions the cellular biophysical environment as a viable and promising therapeutic target for DC.

## Figures and Tables

**Table 1:** The peak positions and assignments of HLECs in the Raman spectra.

Peak Positions (cm^−1^)	Assignments
1006	Phe (protein)
1012	Trp (protein)
1055	–C–N– stretching vibration
1089	–C–C– distortion (lipid)
1130	–C–C– trans (lipid)
1205	Tyr, Phe (protein)
1260	Amine III, random curling (protein)
1308	Amine III, β-folding (protein)
1448	PE, –CH_2_– cleavage (lipid)
1459	–CH_2_– bending; PC, CH2 deformation (lipid)
1660	Amine I (protein)
2878	–CH_2_– anti-symmetric stretching vibration (lipid)
2937	–CH– stretching vibration (lipid, protein)

**Table 2:** Evaluation of S_l_ and S_t_ in glucose-induced HLECs after gigantol treatment using Raman spectroscopy.

Groups	S_l_	Var Rate of S_l_ (%)	S_t_	Var Rate of S_t_ (%)
Control group	0.7287 ± 0.11		0.41002 ± 0.09	
Model group	0.90807 ± 0.056 *	+24.60 (vs. control)	0.51303 ± 0.089 *	+25.10 (vs.)
Gigantol group	0.75949 ± 0.078 **	−19.56 (vs. model)	0.42909 ± 0.058 **	−19.56 (vs.)

* *p*  <  0.05, the model group compared with the control group; ** *p*  <  0.01, the gigantol group compared with the model group.

## Data Availability

The original contributions presented in this study are included in the article. Further inquiries can be directed to the corresponding author.
